# Volunteer Resilience and Burnout: Psychosocial Factors

**DOI:** 10.3390/healthcare14213833

**Published:** 2026-11-09

**Authors:** Einav Levy, Aviva Zrihan Weitzman, Liat Yorav, Yori Gidron, Ayala Cohen

**Affiliations:** 1 Social Work School, Tel Hai Qiryat Shmona University, Kiryat Shmona 1220800, Israel; aweitzma@gmail.com (A.Z.W.); ayalac@telhai.ac.il (A.C.); 2 Social Work Department, Golan Regional Council, Katzrin 1293325, Israel; liaty@megolan.org.il; 3 School of Nursing, Haifa University, Haifa 3103301, Israel; ygidron@univ.haifa.ac.il

**Keywords:** armed conflicts, emergency responders, volunteers, frontline workers, resilience, psychological, community resilience, sense of coherence, self efficacy, anxiety, burnout, psychological

## Abstract

Background: Community-based volunteer emergency teams increasingly serve as frontline responders in disaster-prone and conflict-affected regions, yet little is known about the psychosocial resources sustaining their functioning under prolonged armed conflict. Following the October 2023 escalation of the Israel–Hizballah conflict, Local Emergency Response Team (LERT) volunteers in northern Israel operated under chronic threat and shared collective trauma alongside the communities they served. This study examined how psychosocial resources jointly shape volunteer anxiety and burnout. Methods: A cross-sectional survey of 106 LERT volunteers across 33 communities in northern Israel assessed sense of coherence, self-efficacy, individual resilience, community resilience, emergency training history, anxiety, and burnout, using hierarchical regression analysis. Results: Psychosocial resources were closely interrelated and, together, were linked to lower anxiety, supporting their role as an integrated coping system. Sense of coherence, particularly its comprehensibility component, stood out as the strongest resource linked to lower burnout. Unexpectedly, emergency training was linked to higher anxiety and lower self-efficacy and resilience. Conclusions: Here, we show that volunteer well-being in prolonged conflict depends on an ecology of psychosocial resources spanning individual, relational, and community levels, and that burnout reflects a loss of cognitive coherence rather than exhaustion alone. Training that neglects meaning-making and emotional regulation may unintentionally heighten psychological risk, underscoring the need for psychosocial support in training design, volunteer support, and responder sustainability planning worldwide.

## 1. Introduction

### 1.1. Emergency Volunteering

Emergencies, wars, and disasters are common situations throughout human history. Often, these situations require the intervention of aid workers from various professions such as police, firefighters, paramedics, social workers, and volunteers from a variety of professions who act as first responders (FR) for the victims. These professionals and volunteers often work within the affected area or population and are therefore exposed to a shared traumatic reality (STR) [1,2,3,4]. Baum [1] defined STR in the context of the care professions as a situation characterized by the occurrence of an event that may cause trauma to the entire community to which the helper and the helped belong. The hallmark of this situation is the helper’s dual exposure as an individual member of the affected community and as a professional providing services and care to clients who have also been affected by the traumatic event. The intense demands placed on these FR can generate significant challenges [5].

Globally, there are places where volunteers are organized as community or Local emergency teams (LERTs) and are playing an increasingly critical role in contemporary disaster response systems. LERTs consist of local volunteers trained to offer first aid, emotional support, emergency coordination, and important communication information [6,7,8]. Their role includes offering local knowledge, culturally appropriate communication, and operational adaptability. Originally designed to address short-term crises, these teams provide medical, security, and psychosocial assistance in a variety of emergency situations, often serving as vital intermediaries between affected populations and formal national response mechanisms. Their role is even more critical in geographically dispersed rural communities [9,10].

While some teams receive formal training and gain operational experience in disaster environments, others operate with minimal or no specialized preparation [11].

However, despite the crucial value of FR, empirical evidence demonstrates that high-pressure, unpredictable environments typical of disaster zones can impair team functioning by undermining communication, coordination, information processing, and decision-making processes [12,13]. These vulnerabilities become even more pronounced during extended deployments, such as those occurring in protracted conflicts, pandemics, or cascading disasters. Research across diverse global contexts indicates that such teams frequently encounter coordination breakdowns, resource shortages, and accumulating psychological strain [14,15]. Prolonged exposure to these conditions can foster chronic occupational stress and emotional exhaustion, heightening risks for stress-related symptoms and professional burnout among responders and weakening overall team resilience [16,17]. Ultimately, these constraints diminish operational effectiveness and may compromise both individual well-being and collective performance.

Given Israel’s persistent security challenges, LERTs were established nationwide to address emergency situations. These teams function in communal settlements and municipalities to provide immediate assistance during crises, be they security-related, social, or community-based, until national emergency services arrive [18,19].

The violent conflict between Israel and Hezbollah in northern Israel, which erupted alongside the Israel-Hamas war in Gaza, placed local communities, including those in the northern regional councils, under sustained, proximal threat of missile and drone attacks. While Local Emergency Response Teams (LERTs) in Israel were originally established for short-term interventions, this war exposed them, together with their communities, to a prolonged event lasting approximately two years.

### 1.2. Emotional and Functional Consequences of STR

Prior research has shown that exposure to violent conflict and ongoing security threats is associated with elevated stress, anxiety, depression, and trauma-related symptoms [20,21,22,23]. Within the context of the present study, prolonged exposure to war-related threat is understood as both a source of psychological distress and a challenge to the sustained functioning of community-based emergency responders.

Anxiety frequently emerges as a psychological response in emergency responders, often co-occurring with stress and secondary traumatic symptoms [11]. Characterized by excessive worry and heightened physiological arousal, such as alertness, rapid heartbeat, muscle tension, and cognitive impairments like difficulty concentrating, anxiety can significantly interfere with performance in high-stakes environments [24]. In emergency contexts, such anxiety typically stems from the anticipation of danger, uncertainty about outcomes, or moral injury. Anxiety often co-occurs with other responses such as hypervigilance, sleep disturbances, and emotional numbing [25]. Chronic anxiety may compromise clinical and operational judgment, thereby increasing the risk of errors under pressure [26]. Research indicates that untreated anxiety can escalate and serve as a precursor to post-traumatic stress disorder (PTSD). Berger et al. [27] found significantly higher rates of PTSD and anxiety-related disorders among rescue workers compared to the general population, with paramedics in Asia particularly vulnerable. Similarly, a meta-analysis by Petrie et al. [28] confirmed elevated rates of anxiety and trauma-related symptoms among ambulance personnel, especially following exposure to mass-casualty events. Comparable patterns were observed in Brazil, Italy, and elsewhere during public health emergencies, where persistent threat and exposure to suffering contributed to elevated anxiety and psychological strain among aid personnel [29].

Unlike anxiety, which may emerge as a more immediate emotional response to threat, burnout develops gradually through sustained overload, insufficient recovery opportunities, role strain, and the erosion of meaning. Defined as a prolonged psychological response to chronic occupational and interpersonal stressors, burnout manifests through emotional exhaustion, cynicism, depersonalization, and reduced professional efficacy [30,31]. Among emergency responders and aid workers, this condition is exacerbated by repeated exposure to suffering, unpredictable work demands, limited organizational support, and insufficient opportunities for psychological recovery [32,33,34]. Within STR, the risk of burnout may be particularly pronounced; the dual role eliminates psychological distance and limits opportunities for restoration [5]. Recent studies further indicate that burnout is highly prevalent among emergency medical personnel and may impair both responders’ mental health and the quality of care provided under crisis conditions [35,36].

Yet the effects of prolonged exposure are not uniform. Personal, relational, and community resources may mitigate the emotional and functional toll of emergencies and support adaptation over time [34,37,38]. However, it can be argued that without appropriate support mechanisms, including psychological first aid, supervision, rest protocols, and mental health interventions, FR may experience deteriorating functioning over time, ultimately compromising the quality and sustainability of response efforts [39]. Accordingly, the present study examines anxiety and burnout alongside the ecology of psychosocial resources that may sustain LERT members’ functioning under prolonged war-related threat.

### 1.3. Ecology of Resilience Factors: Psychosocial Resources

Resilience refers to the capacity to adapt, persist, and recover in the face of adversity. Contemporary resilience literature increasingly conceptualizes resilience not only as an individual trait, but as a social-ecological process shaped by the availability of supportive relationships, meaningful roles, and accessible resources in the surrounding environment [40,41,42]. An ecological approach emphasizes individuals’ capacity to navigate toward and negotiate for the resources required to sustain well-being during adversity, alongside the environment’s capacity to provide those resources in contextually meaningful ways [43]. Consequently, under conditions of chronic stress, emergencies, and war, a dynamic spectrum of psychosocial protective factors supports coping—ranging from personal attributes like a sense of coherence [44] and self-efficacy [45], to interpersonal dynamics and community-level capacities [37,46]. Rather than operating in isolation, these multi-level resources continuously interact across individual, team, and systemic domains, shaping both emotional responses, such as anxiety, and functional outcomes, including burnout [34,47].

At the macro-level of this social-ecological matrix, Community Resilience refers to the capacity of a community to withstand, adapt to, and recover from disasters or prolonged emergencies through collective resources, social networks, leadership, preparedness, and mutual trust [9,46,48,49]. Reaching beyond the aggregation of individual coping capacities, this is a collective resource embedded in community structures, relationships, and institutions. Cohen et al. [48] identified five interrelated components of community resilience: leadership, collective efficacy, preparedness, place attachment, and social trust, reflecting a community’s perceived capacity to organize, support its members, maintain belonging, and sustain trust. This framework is supported by studies showing that coping patterns vary according to community type and available resources; for instance, Braun-Lewensohn and Sagy [50] found that urban residents reported higher stress and weaker community resources compared to rural residents, relying more heavily on personal coping assets. For Local Emergency Response Teams (LERTs), community resilience is particularly critical, as their operational capacity depends not only on personal resources but also on the structural strength, cohesion, and readiness of the community as a whole [21]. Thus, community resilience serves as a key protective psychosocial factor associated with responders’ emotional well-being and sustained emergency functioning under prolonged threat.

At the micro-level of this ecological framework, personal psychosocial resources play a pivotal role in enabling individuals to evaluate stress, maintain psychological stability, and actively mobilize external supports. Prominent among these resources are a Sense of Coherence (SOC) [44], Self-Efficacy [45], and Individual Resilience as conceptualized by Ungar [41,43]. While SOC and self-efficacy provide the cognitive and motivational foundation for coping, individual resilience reflects the functional capacity of personnel to navigate toward and negotiate for psychological, social, and structural assets under acute stress.

Sense of Coherence (SOC) refers to a global orientation through which individuals perceive life events and demands as comprehensible, manageable, and meaningful [44]. In line with the salutogenic model, SOC serves as a key interpretive resource in emergency and disaster contexts. Its three core dimensions—comprehensibility, manageability, and meaningfulness—enable individuals to make chaotic operational experiences intelligible, identify available coping resources across their environment, and sustain motivation under severe pressure [38,44,51]. Consequently, higher SOC is consistently linked to lower stress, psychological distress, and burnout, particularly in contexts marked by chronic uncertainty and threat [38,52,53], aligning with research demonstrating that personal meaning serves as a vital buffer against operational strain [34].

Complementing SOC’s global orientation, Self-Efficacy refers to individuals’ belief in their capacity to organize and execute specific actions required to manage demanding tasks [45]. In disaster and emergency settings, self-efficacy becomes critical as responders operate under extreme uncertainty, time pressure, and personal risk. Higher self-efficacy promotes proactive stress management, sustained effort, adaptive coping, and heightened crisis readiness [54,55,56]. Furthermore, strong efficacy beliefs enhance personnel’s perceived capacity to execute mission-critical tasks, reducing vulnerability to trauma-related symptoms and functional impairment [57]. Together, community resilience alongside Sense of Coherence, Self-Efficacy, and Individual Resilience can function as interconnected anchors within the social-ecological matrix, co-occurring with lower acute anxiety and reduced long-term burnout among the team.

The current cross-sectional study examines the emotional and functional consequences of extended emergency volunteering among LERT members operating under these conditions of sustained threat and reality (STR), focusing specifically on anxiety and burnout. It contributes to the literature by being the first to examine how macro-level community resilience and micro-level personal assets—namely sense of coherence (SOC), self-efficacy, and individual resilience—co-occur and interrelate to account for these outcomes among volunteer responders enduring prolonged shared trauma. The study further accounts for key background characteristics, including length of residence and specialized emergency training. A comprehensive examination of this social-ecological matrix is essential for developing evidence-based interventions that preserve mental health, mitigate burnout, and sustain the well-being of emergency volunteers during extended crises.

Grounded in this social-ecological framework and acknowledging the correlational design of this cross-sectional study, the following hypotheses were formulated:

**H1a.**  *Community resilience, SOC, self-efficacy, and individual resilience will be negatively associated with anxiety levels among LERT members.*

**H1b.**  *Community resilience, SOC, self-efficacy, and individual resilience will be negatively associated with burnout levels among LERT members.*

**H2.**  *In hierarchical multiple regression models, personal and community psychosocial resources will account for significant additional variance in anxiety and burnout beyond demographic, role-related, and exposure characteristics.*

## 2. Method

This study employed a cross-sectional survey design to examine protective and risk resources for anxiety and burnout among LERT volunteers. The study was conducted in 33 communities in the northern regional council of Israel between March 2025 and July 2025.

### 2.1. Procedure

Following ethical approval provided by the University IRB committee of the University (approval no. (24-14-9)), a call for participants was published through the northern regional council. Eligible participants were required to hold an active LERT position during the study period, be 18+, and reside in one of the 33 communities. No additional exclusion criteria were applied. Regional council personnel records identified 264 eligible LERT volunteers. Each record represented a unique individual holding a single LERT position; no volunteer held more than one position, and similar roles in different communities were held by different volunteers. All 264 eligible volunteers received a direct invitation to participate. Participants received an anonymized online questionnaire including a description of the study’s goal and a consent form, which they completed voluntarily prior to participation. The data were collected by Google Forms, and since participants had to complete each item in order to move to the next item, there were no missing data.

### 2.2. Participants

One hundred and six volunteering LERT members participated in the study out of the 264 invited volunteers, yielding a response rate of 40.15%, favorable compared to similar studies (15–20% and 44.1%, respectively) [58,59]. We conducted a post hoc sample-size analysis. Assuming 2 significant background predictors, testing the contribution of 4 additional predictors that explain 15% of the variance in each outcome, assuming statistical power of 0.80 and statistical significance of *p* < 0.05, 85 participants would be required. The age range was 30–75 years, and the majority of participants were women (60.2%).

### 2.3. Measures

Demographic variables included age, gender, family status, having children and their ages, religiosity, residential location, number of years in the place of residence, job during routine times, job during the war, roles during emergency times, number of years in the role, and whether they underwent training during the war.

All LERTs are monitored and supervised by the social services of the regional council; thus, the training courses were similar between all the LERTs and included content relevant to the volunteers’ role.

Psychological variables include SOC, self-efficacy, resilience, and community resilience as independent variables. Burnout and anxiety were defined as outcome measures.

To quantify SOC, Antonovsky [44] developed the Orientation to Life Questionnaire, available in a 29-item version (SOC-29) and a shorter 13-item version (SOC-13). The SOC-13 scale comprises three components: Comprehensibility (items 2, 6, 8, 9, 11), Manageability (items 3, 5, 10, 13), and Meaningfulness (items 1, 4, 7, 12). Each item is rated on a 7-point semantic differential scale, with response anchors varying by item (e.g., ranging from ‘never’ to ‘very often’, or from ‘no clear goals’ to ‘very clear goals’), such that higher scores reflect a stronger sense of coherence. A total SOC score is computed by summing responses across all 13 items, and subscale scores are computed by summing the respective items listed above, with higher scores indicating greater perceived comprehensibility, manageability, or meaningfulness. These instruments have been validated across diverse populations and cultural contexts. Notably, while Antonovsky viewed SOC as a unidimensional construct, subsequent research suggests a multidimensional structure, with each component contributing uniquely to the overall sense of coherence [60]. In this research, we used the 13-item scale to reduce the burden on participants and the risk of attrition. The Cronbach alpha of the scale in the current study was 0.83.

Self-efficacy was assessed through the eight-item New General Self-Efficacy (NGSE) scale [61]. Each item is rated on a 5-point Likert scale ranging from 1 (‘strongly disagree’) to 5 (‘strongly agree’), with three reverse-scored items. A total score is computed as the mean of the eight items, with higher scores reflecting greater generalized self-efficacy. This scale has good psychometric properties and predicts task-specific self-efficacy, as validated in multicultural contexts [62]. The Cronbach’s alpha of the scale in the current study was 0.91.

Resilience was assessed using the Adult Resilience Measure Revised (ARM-R) [42,63,64]. Click or tap here to enter text., which consists of 17 Likert-type items. Respondents indicated on a five-point scale to what extent the statement was true for them. Higher scores indicate a greater presence of (and engagement with) supportive resources in an individual’s environment, indicative of an individual’s resilience. These social-ecological resources include a sense of belonging in one’s community, family and peer support, and having opportunities to apply one’s abilities. The total resilience score was calculated by summing each of the items.

In the current study, the ARM-R scale showed high internal consistency, with a Cronbach’s alpha of 0.91.

Community resilience was assessed using the short, 10-item version of the Conjoint Community Resilience Assessment Measure (CCRAM-10) [48,65], a self-report instrument comprising five factors: collective efficacy, preparedness, social trust, place attachment, and leadership (the latter reflecting respondents’ faith in and trust toward local leaders and their perceived ability to lead change). All items were rated on a 5-point Likert scale (1 = Disagree, 5 = Strongly Agree), and an average score was calculated for all items. The Cronbach’s alpha of the scale in the current study was 0.88.

Burnout was assessed using the 15-item Maslach Burnout Inventory–Student Survey (MBI-SS) [66], derived from the original Maslach Burnout Inventory [67], in its Hebrew version [68]. The instrument assesses three dimensions of burnout: emotional exhaustion, cynicism, and reduced efficacy. Item wording was adapted to refer to volunteer activity in the emergency team rather than to studies. Respondents rated how often they experienced each feeling on a 7-point frequency scale ranging from 0 (never) to 6 (every day). Efficacy items were reverse-scored, and a total burnout score was computed as the mean of the 15 items (range 0–6), with higher scores indicating greater burnout. The Hebrew version showed good reliability among Israeli respondents [68]. The Cronbach alpha of the scale in the current study was 0.83.

Anxiety was assessed using the State scale of the State-Trait Anxiety Inventory (STAI) [69], employing the six-item short form developed by Marteau and Bekker [70]. The short form was derived from the original 20-item State scale using item-remainder correlations to select the six items most highly correlated with the total score, evenly split between anxiety-present and anxiety-absent items, and has been shown to produce scores highly comparable to the full-form STAI while reducing respondent burden. Items are rated on a 5-point Likert scale ranging from 1 (‘not at all’) to 5 (‘very much so’) and reflect how the respondent ‘typically feels’. Research has demonstrated that the short-form STAI has adequate concurrent validity and internal consistency relative to the full-form scale [71]. The Cronbach alpha of the scale in the current study was 0.80.

### 2.4. Statistics

The first stage of data analysis involved descriptive statistics for the study variables, including means and standard deviations for continuous variables and percentages for categorical variables. The second stage examined correlations between the key research variables (SOC, self-efficacy, resilience and community resilience) and anxiety and burnout) and group comparison tests using *t*-tests when comparing the trained and the untrained participants.

After finding significant background correlates of anxiety and burnout, we conducted a hierarchical multiple linear regression testing whether SOC, resilience and community resilience account for additional variance in each outcome (block 2) after controlling for significant background correlates (block 1). We used a statistical significance cut-off of *p* < 0.05 and SPSS 27.0 software.

For two comparisons where directional hypotheses were specified a priori on the basis of prior literature, one-tailed significance tests were used; all other tests were two-tailed. First, the hypothesized positive association between anxiety and burnout was based on extensive literature documenting the co-occurrence of these outcomes in high-stress, professional contexts.

## 3. Results

As seen in Table 1, most participants were women, married, secular, and had undergone emergency training. Background correlates of anxiety and burnout were examined first (see Table 1 for full statistics). Age and years of residence were both negatively associated with burnout. Anxiety was associated with years of residence but not with age or gender. Both religiosity and gender were unrelated to any outcome.

As shown in Table 2 and Table 3, the four psychosocial resources were largely intercorrelated in the expected direction: self-efficacy was positively associated with SOC and individual resilience, but contrary to expectation, it was unrelated to community resilience. SOC and community resilience were each positively associated with individual resilience.

As shown in Table 4, all four psychosocial resources were negatively associated with anxiety; all except individual resilience were also negatively associated with burnout. Anxiety and burnout were themselves positively correlated with one another (see Table 4).

We then explored the effects of training on the variables. Compared with volunteers who had not undergone emergency training, trained volunteers reported significantly higher anxiety and significantly lower individual resilience and self-efficacy. Training status was unrelated to SOC and community resilience.

### Multivariate Analysis

Because age and years of residence were highly correlated (see Table 1), and because years of residence is more directly related to embeddedness in community life, only years of residence was retained in the regression models. We used hierarchical multiple regression to explain variance separately for anxiety and burnout. In these regressions, we entered years of residence and training status in Block 1 and community resilience, self-efficacy, and SOC in Block 2. Regression diagnostics indicated that the assumption of multicollinearity was met, but the assumption of homoscedasticity was not met for either outcome (see limitations below). Importantly, when correcting the heteroscedasticity by the weight estimation method and using the Weighted Least Squares calculation (we selected SOC since it had the highest SD), the explained variance did not change for burnout and slightly improved for anxiety; thus, we present the results without these corrections.

After controlling for years of residence and training, community resilience, self-efficacy, and SOC together accounted for a significant increment in explained variance in burnout, with SOC and specifically its comprehensibility dimension, emerging as the only significant correlate in the final model (see Table 5 for full regression statistics). For anxiety, the same set of psychosocial resources also accounted for a significant increment in explained variance beyond years of residence and training; however, in the final model, training status remained the only significant correlate (see Table 5).

Taken together, most psychosocial resources were positively intercorrelated and negatively associated with anxiety. Regarding burnout, only SOC, specifically its comprehensibility dimension, remained significant in the multivariable model. Emergency training was associated with higher anxiety and lower resilience and self-efficacy, but was unrelated to SOC and community resilience. Table 5 summarizes these associations.

## 4. Discussion

This study examined the psychosocial resources that protect the mental well-being and functioning of Local Emergency Response Team (LERT) members under prolonged, active conflict. The findings indicate that these resources shape two distinct outcomes, anxiety and burnout, through different mechanisms: sense of coherence (SOC) and self-efficacy were broadly associated with lower anxiety, whereas among functional outcomes only SOC’s comprehensibility component remained significantly associated with burnout, and emergency training was associated with higher rather than lower anxiety. Given the cross-sectional design, all associations reported below describe patterns observed at a single point in time rather than confirmed causal or protective effects.

This study was conducted during a period of sustained political violence beginning with the October 2023 terror attack on Israel and continuing through roughly a year and a half of regional conflict, during which LERTs functioned not as external responders but as community members exposed to the same ongoing threat they were mobilized to address.

Bivariate analyses indicated that most psychosocial resources were positively associated with one another, consistent with their functioning as an interconnected coping system rather than isolated assets: self-efficacy was associated with SOC and personal resilience, SOC was associated with both personal and community resilience, and community resilience was associated with personal resilience, underscoring the interplay between individual- and community-level resources in line with conceptions of resilience as a systemic, ecological process [41]. No significant association emerged between self-efficacy and community resilience, reinforcing the conceptual distinction between individual coping beliefs [45] and collective resources embedded in social trust, infrastructure, leadership, and community readiness [9,49]. Within this system, SOC appears to function as a core asset bridging internal coping capacities and the perception of resources available in the community environment, consistent with the salutogenic framework [44], in which SOC enables individuals to understand a stressor, perceive that they possess the resources to manage it, and locate meaning within the coping process; a systematic review similarly identifies SOC as a statistically significant protective variable associated with fewer negative mental health symptoms [72,73]. Accordingly, all examined psychosocial resources were negatively associated with anxiety, consistent with a broadly protective role against emotional distress in ongoing emergency conditions.

Regarding burnout, a more distinct and complex pattern emerged. Although self-efficacy, SOC, and community resilience were each negatively associated with burnout at the bivariate level, only SOC remained significantly associated with burnout in the multivariate model, consistent with other findings [73]. Analysis of SOC’s component structure indicated that comprehensibility, the capacity to perceive reality as organized, predictable, and understandable, was the component primarily accounting for this association. This suggests that burnout in contexts of prolonged emergency reflects not only emotional exhaustion or depleted energy but a deeper disruption of cognitive coherence and meaning-making. Burnout has similarly been linked to declines in executive function, attention, and memory, particularly in professions combining high work pressure with high cognitive demand [74]; when reality is perceived as chaotic, unpredictable, and lacking internal logic, cognitive load may become a central driver of the burnout process, consistent with views of burnout as a process of prolonged overload and eroding meaning rather than simple emotional depletion [31,75]. Under these conditions, the critical resource for LERT members may not be the capacity to bounce back or simply continue functioning, but the capacity to maintain a comprehensible, organized, and meaningful account of an unpredictable reality, which may also explain why personal resilience was not significantly associated with burnout in the multivariate model: resilience may sustain continued functioning and perseverance within an ongoing shared traumatic reality without necessarily preventing burnout that results from chronic uncertainty, blurred boundaries between role and personal life, and a diminished capacity to organize the experience.

The regression model for anxiety pointed to a different pattern. After accounting for background characteristics and psychosocial resources, emergency training was the only variable significantly associated with anxiety in the multivariate model. Contrary to the expectation that training would function protectively, it was associated with higher anxiety and with lower self-efficacy and resilience. This paradoxical pattern is consistent with a broader tension identified in the military and first-responder psychology literature: technical, scenario-based training that rehearses threat exposure can heighten situational threat awareness and anticipatory hypervigilance when it is not paired with components that support emotional regulation, cognitive processing, and meaning-making [11,39]. Stress-inoculation and exposure-based training models developed for military and tactical populations similarly indicate that repeated rehearsal of threat scenarios can sharpen risk appraisal and sustain physiological arousal unless deliberately paired with regulation-focused debriefing and cognitive-reframing components [28,29]; in the absence of such components, training may function as repeated exposure to threat cues rather than as a source of mastery and reassurance. Applied to the present sample, this suggests that LERT training—typically oriented toward operational and technical competence—may increase threat salience without offsetting it through structured psychological containment, leaving trained members more, rather than less, anxious. This interpretation should, however, be weighed against plausible alternative accounts: because the design is cross-sectional, it is equally possible that individuals with higher baseline anxiety self-select into training, or that trained and untrained members differ in unmeasured ways—such as exposure intensity, role, or organizational context—that independently affect anxiety. Longitudinal data tracking individuals before and after training would be needed to distinguish these possibilities from a genuine training effect.

The positive association between anxiety and burnout further suggests a pattern consistent with a reciprocal relationship, in which the two may co-occur and reinforce one another over time, a possibility that would require longitudinal data to confirm. The differential pattern of associations for each outcome nonetheless points to distinct underlying mechanisms: anxiety appears most closely tied to situational exposure and heightened alertness, whereas burnout is more closely tied to disruptions in cognitive coherence and meaning-making. In this sense, burnout may represent a longer-term functional outcome of sustained meaning erosion rather than an immediate emotional response to threat.

From an ecological perspective, these findings indicate that the psychosocial functioning of first responders is shaped across several interacting levels. At the micro-level, SOC and self-efficacy function as interpretive and regulatory mechanisms shaping how stress is perceived and managed. At the relational and team level, resilience and burnout develop through ongoing interactions under pressure. At the community level, community resilience reflects trust, readiness, leadership, and collective efficacy, shaping the extent to which teams feel supported and contained. Resilience, in this framework, is not merely an internal trait but a dynamic process generated at the intersection of the individual, the team, the community, and the surrounding organizational structures [41].

Building on these findings, and keeping in mind this study was a cross-sectional design, we nevertheless propose a hypothetical conceptual path model (Figure 1) to organize the observed cross-sectional associations into a coherent framework for future testing; it should be read as a set of relationships derived from the pattern of associations found here, not as an established explanatory or causal pathway. In this model, psychosocial resources are positioned as interconnected correlates with differentiated associations: SOC and self-efficacy are associated with lower anxiety and burnout, while individual resilience alone is not significantly associated with burnout in this sample. SOC, and particularly its comprehensibility component, is proposed as a candidate mechanism warranting longitudinal investigation, and emergency training is proposed as associated with higher anxiety in a pattern that may reflect training intensifying anxiety when it is not accompanied by emotional-regulation and cognitive-containment components, though this directionality cannot be confirmed with the present design.

Age and years of residency were associated with lower burnout, underscoring the potential importance of accumulated experience, contextual familiarity, and community embedding. Consistent with prior research, older participants reported lower stress and higher self-efficacy than younger participants, which may reflect both long-term experience coping with adversity and the accumulation of spiritual, mental, and emotional resources over time [76]. Because these variables cannot be directly modified, their practical significance lies in pointing toward the resources that can be: sense of coherence, self-efficacy, emotional regulation skills, and meaning-making capacity. Team composition, seniority, and continuity of community connection should accordingly be treated as components of structural risk management rather than purely administrative considerations.

Taken together, these findings indicate that psychosocial sustainability must be treated as a core design criterion in decentralized emergency systems, and specifically in the training of LERT members: technical competence is a necessary but not sufficient condition for the sustained functioning of volunteer responders, and individual, team, and community resources must be developed in parallel to support long-term functioning and well-being. Because emergency training was associated with higher anxiety, training programs should be evaluated not only for operational skill acquisition but for their psychosocial impact, incorporating components such as emotional regulation, structured debriefing, processing of uncertain situations, reinforcement of self-efficacy, and the construction of meaning and coherence, potentially reducing excessive hypervigilance while strengthening readiness. Because comprehensibility was the SOC component most closely associated with burnout, mitigating burnout is likely to require more than general emotional support: teams need help structuring the reality in which they operate, through organizational clarity, distinct role definitions, consistent communication routines, transparent decision-making, predictable information flow, and visible leadership; these elements may plausibly serve as structural buffers against cognitive burnout, pending confirmation through prospective research. The protective association of community embedding further suggests that recruitment, retention, and team composition function as structural risk-management levers: combining veteran and newer volunteers, establishing intergenerational mentoring, and maintaining team continuity may strengthen stability, organizational learning, and functional resilience over time. Finally, implementing brief, periodic monitoring of anxiety, burnout, and perceived clarity, paired with predetermined support pathways, could allow local authorities and emergency managers to identify cumulative overload early, intervene before functional decline or dropout, and treat responder well-being as an integral component of operational readiness.

Although this study was conducted within the specific context of the Israel–Hezbollah conflict, its theoretical and practical implications extend to disaster risk reduction systems more broadly. Community-based volunteer emergency teams operate across many low- and middle-income countries, fragile states, and disaster-prone regions where formal professional response capacity is limited or delayed. The ecological framework applied here, integrating individual resources (SOC, self-efficacy), relational resources (individual resilience), and community-level resources (community resilience), offers a transferable lens for understanding volunteer sustainability across protracted emergency settings, including floods, earthquakes, pandemics, and complex humanitarian crises. That SOC, and specifically its comprehensibility dimension, was the resource most closely associated with burnout has direct relevance for volunteer training design worldwide: it suggests that operational training alone may be insufficient to support responder well-being, and that structural clarity, role definition, and meaning-making need to be embedded in emergency preparedness systems as standard components. Similarly, the paradoxical association between training and elevated anxiety invites reexamination of training paradigms in international disaster response frameworks. By situating local volunteer functioning within an ecological, community-resilience perspective, this study contributes empirical grounding to Sendai Framework priorities concerning the strengthening of local disaster risk governance and the protection of frontline responders, with relevance to DRR policy and volunteer emergency programs across diverse national and cultural contexts.

Several limitations should be considered when interpreting these findings. First, and most centrally, the cross-sectional correlational design does not permit causal inference: it cannot establish whether participation in training increased anxiety, or whether individuals with higher baseline anxiety were more inclined to seek out training, and longitudinal studies, particularly those assessing participants before and after training, are needed to clarify the directionality of these associations and the long-term effects of training. Second, the sample was geographically concentrated among LERT members in northern Israel under conditions of ongoing security exposure; while this context enabled examination of functioning within a shared traumatic reality, it may limit generalizability to other regions, cultures, and community structures, and the sample’s relatively high proportion of women, married participants, and secular participants warrants caution in interpretation, as patterns of community resilience and psychosocial resources may operate differently across demographic and religious groups. Third, the study did not assess the content, quality, or type of training received, making it impossible to determine which specific training components were associated with higher anxiety and lower self-efficacy and resilience, a gap that bears directly on the training-related interpretation offered above and should be a priority for future data collection.

In sum, this study advances an ecological understanding of protective resources among LERTs, showing that self-efficacy, SOC, and individual and community resilience function as an interdependent coping system rather than isolated traits, with SOC, and particularly its comprehensibility component, emerging as the resource most closely associated with reduced burnout under prolonged, unpredictable threat. Practically, these findings argue for redesigning LERT training to address psychosocial as well as operational readiness: embedding structured debriefing, role clarity, predictable communication routines, and meaning-making support, while critically reexamining why standard training was paradoxically associated with heightened anxiety. Several methodological issues remain unresolved: the cross-sectional design precludes causal inference about training, resilience, and distress; the sample was geographically concentrated and demographically skewed toward secular, married, female volunteers in a single conflict context; and training content and quality were not assessed. Future research should prioritize multicenter designs spanning different disaster types and response systems, longitudinal studies tracking volunteers before, during, and after training and deployment, intervention trials testing coherence- and clarity-based training modules, and culturally diverse samples across varied community structures and conflict or disaster settings, to test whether this ecological model generalizes beyond the present context. As natural and human-made disasters increase globally, so too does reliance on local volunteer responders, making the sustained protection of their psychosocial well-being a core component of disaster preparedness rather than a peripheral concern.

## Figures and Tables

**Figure 1:**
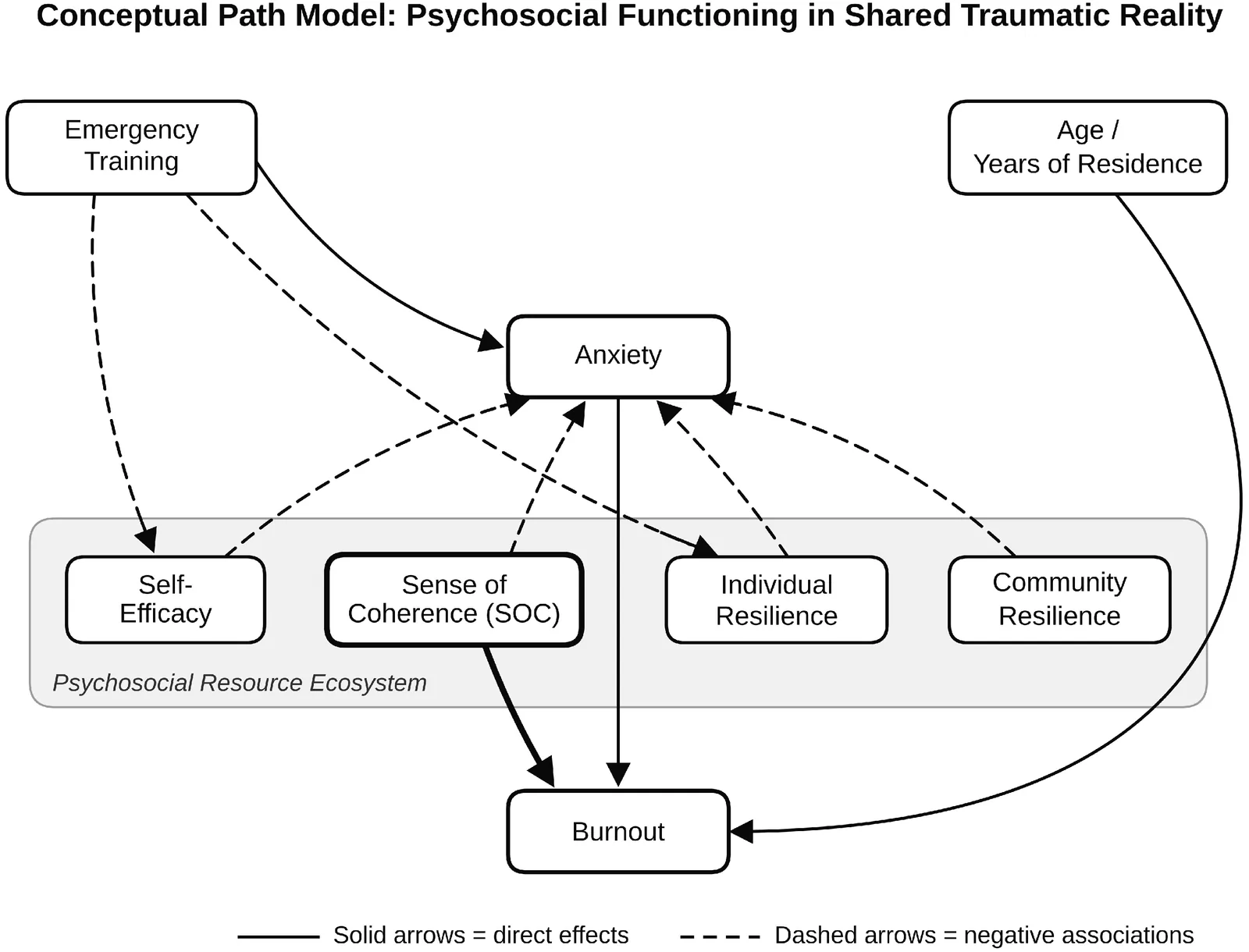
The Ecology of Protectiv.

**Table 1:** Demographic variables.

	Percentage
**Gender**	
Men, Women	38.9%, 60.2%
**Personal status**	
Widow	0.9%
With partner	2.8%
Divorced	7.4%
Married	86.1%
Single	1.9%
**Religiosity**	
Secular	64.8%
Traditional	10.2%
Religious	21.3%
Ultra-Orthodox	0.9%
Emergency training	61.1%

**Table 2:** Main study variables.

	Mean	SD
Self—Efficacy	49.49	6.47
Sense of Coherence	71.46	9.95
Individual resilience	71.46	7.61
Community resilience	39.19	4.67
Anxiety	14.21	4.04
Burnout	35.78	11.00

**Table 3:** Variables Correlation.

	SOC	Individual Res.	Community Res.
Self-Efficacy	0.320 *	0.658 *	0.61
SOC	1	0.218 *	0.369 *
Individual Res.	0.218 *	1	0.212 *
Community Res.	0.369 *	0.212 *	1

* *p* < 0.001.

**Table 4:** Outcomes Correlation.

	Self-Efficacy	SOC	Individual Res.	Community Res.
Anxiety	−0.295 *	−0.292 *	−0.306 *	−0.201 *
Burnout	−0.241 *	−0.410 *	−0.110	−0.324 *

* *p* < 0.001.

**Table 5:** Associations Between Psychosocial Variables, Anxiety, and Burnout.

Psychosocial Variable	Association with Anxiety	Association with Burnout
Self-efficacy	Significant negative	Significant negative
SOC	Significant negative	Strongest correlate
Individual resilience	Significant negative	No significant association
Community resilience	Significant negative	Significant negative
Emergency training	Significant positive	No significant association
Years of residence	Significant negative	Significant negative

## Data Availability

The data presented in this study are available on request from the corresponding author due to ethical considerations.
